# ZZ-Type a posteriori error estimators for adaptive boundary element methods on a curve^☆^

**DOI:** 10.1016/j.enganabound.2013.10.008

**Published:** 2014-01

**Authors:** Michael Feischl, Thomas Führer, Michael Karkulik, Dirk Praetorius

**Affiliations:** a Institute for Analysis and Scientific Computing, Vienna University of Technology, Wiedner Hauptstr. 8-10/E101/4, 1040 Wien, Austria; b Facultad de Matemáticas, Pontificia Universidad Católica de Chile, Avenida Vicuña Mackenna 4860, Santiago, Chile

**Keywords:** Boundary element method, Local mesh-refinement, Adaptive algorithm, ZZ-type error estimator

## Abstract

In the context of the adaptive finite element method (FEM), ZZ-error estimators named after Zienkiewicz and Zhu (1987) [52] are mathematically well-established and widely used in practice. In this work, we propose and analyze ZZ-type error estimators for the adaptive boundary element method (BEM). We consider weakly singular and hyper-singular integral equations and prove, in particular, convergence of the related adaptive mesh-refining algorithms. Throughout, the theoretical findings are underlined by numerical experiments.

## 1 Introduction

Since the seminal work of Zienkiewicz and Zhu [52], averaging techniques became popular in engineering and applied sciences for the a posteriori error control of the finite element solution of partial differential equations. To sketch the idea, we consider the most simple context of the 2D Poisson equation

(1) −Δu=finΩ,u=0on∂Ω.

Here and throughout the work, $\Omega \subset R^{2}$ is a bounded Lipschitz domain with polygonal boundary ∂Ω.

Let $T_{h}$ denote a regular triangulation of Ω into compact, nondegenerate triangles. Let $P^{0}(T_{h})$ be the space of all $T_{h}-\mathrm{piecewise}$ constant functions and $S^{1}(T_{h})$ be the space of all $T_{h}-\mathrm{piecewise}$ affine and globally continuous splines. The lowest-order finite element solution $u_{h}\in S_{0}^{1}(T_{h})\;≔\{v_{h}\in S^{1}(T_{h}):v_{h}=0\;\mathrm{on}\;\partial \Omega \}$ is the unique solution of the Galerkin formulation

(2) $$\int_{\Omega }\nabla u_{h}\cdot \nabla v_{h}\;\mathrm{dx}=\int_{\Omega }{\mathrm{fv}}_{h}\;\mathrm{dx}$$

for all test functions $v_{h}\in S_{0}^{1}(T_{h})$. In this context, the ZZ error estimator reads

(3) $$\eta_{h}=\|(1-A_{h})\nabla u_{h}{\|}_{L^{2}(\Omega )},$$

where $A_{h}:P^{0}{\left(T_{h}\right)}^{2}\to S^{1}{\left(T_{h}\right)}^{2}$ is some averaging operator which maps the $T_{h}-\mathrm{piecewise}$ constant gradient $\nabla u_{h}\in P^{0}{\left(T_{h}\right)}^{2}$ onto some continuous and piecewise affine function $A_{h}\nabla u_{h}\in S^{1}{\left(T_{h}\right)}^{2}$. Possible choices for $A_{h}$ are the usual Clément-type operators like

(4) $$(A_{h}v)(z)=\frac{1}{\mathrm{area}(\omega_{z})}\int_{\omega_{z}}v\;\mathrm{dx}$$

for all nodes $z\in K_{h}$ of $T_{h}$, where

(5) $$\omega_{z}\;\;≔⋃\{T\in T_{h}:z\in T\}$$

denotes the patch of *z*, i.e., the union of all elements $T\in T_{h}$ which have *z* as a node. Although ZZ error estimators are strikingly simple and mathematically well-developed for the finite element method, see e.g., [5,6,11,40], they have not been considered for boundary element methods, yet.

Numerical analysis of adaptive BEM was initiated by the pioneering works [48–51]. By now, available error estimators from the literature include residual-based error estimators for weakly singular [19,20,10,12,15,26,27] and hyper-singular integral equations [20,10,14], hierarchical error estimators for weakly singular [25,32,39] and hyper-singular integral equations [31,32], (*h*−*h*/2)-based error estimators [24,23,29], averaging on large patches [16–18], and estimators based on the use of the full Calderón system [21,22,34,37,41,43]. The reader is also referred to the overviews given in [12,23,35] and the references therein.

This note proposes ZZ-type error estimators in the context of the boundary element method. As model problems serve the hyper-singular and the weakly singular integral equation associated with the 2D Laplacian. Difficulties arise from the fact that neither the involved integral operators nor the energy norms are local.

The outline of this paper reads as follows: In Section 2, we consider the hyper-singular integral equation, introduce a ZZ-type error estimator, and provide numerical evidence for its successful use on a slit model problem as well as for the first-kind integral formulation of some Neumann problem. In Section 3, we apply this approach in the context of the weakly singular integral equation. While Sections 2 and 3 are written for a general audience, Section 4 collects the preliminaries for the numerical analysis of the proposed a posteriori error estimators. A rigorous a posteriori error analysis is postponed to Section 5. The final Section 6 even proves convergence of the standard adaptive mesh-refining algorithm steered by the ZZ-type error estimators proposed.

## 2 Hyper-singular integral equation

We suppose that $\Omega \subset R^{2}$ is simply connected, i.e., Ω has no holes and ∂Ω thus is connected. We denote the fundamental solution of the 2D Laplacian by

(6) $$G(z)\;\;≔-\frac{1}{2\pi }\log|z|\;\mathrm{for}\;z\in R^{2}â§¹\{0\}.$$

Let Γ be some relatively open and connected subset of the boundary ∂Ω. Then, the hyper-singular integral operator is formally defined by

(7) $$\left(\mathrm{Wu})(x)=-\partial_{n(x)}\int_{\Gamma }\partial_{n(y)}G(x-y)u(y)\;d\Gamma (y\right)$$

for x∈Γ. Here, $\int_{\Gamma }\;d\Gamma$ denotes integration along the curve and $\partial_{n(x)}$ is the normal derivative at some point x∈Γ. The hyper-singular integral equation reads

(8) Wu=fonΓ.

For the following facts on the functional analytic setting as well as for proofs and further details, the reader is referred to, e.g., the monographs [33,36,42].

### 2.1 Slit model problem

Assume that Γ⫋∂Ω is not closed. Let ${\overset{˜}{H}}^{1/2}(\Gamma )$ denote the space of all $H^{1/2}(\Gamma )-\mathrm{functions}$ which vanish at the tips of Γ. Then, *W* is a linear, bounded, and elliptic operator between the fractional-order Sobolev space ${\overset{˜}{H}}^{1/2}(\Gamma )$ and its dual space $H^{-1/2}(\Gamma )$, where duality is understood with respect to the extended $L^{2}(\Gamma )-\mathrm{scalar}$ product ${\langle \cdot ,\cdot \rangle }_{L^{2}(\Gamma )}$. Let $f\in H^{-1/2}(\Gamma )$. The variational form of (8) reads

(9) $${\langle \mathrm{Wu},v\rangle }_{L^{2}(\Gamma )}={\langle f,v\rangle }_{L^{2}(\Gamma )}\;\mathrm{for}\;\mathrm{all}\;v\in {\overset{˜}{H}}^{1/2}(\Gamma ).$$

Since the left-hand side defines a scalar product on ${\overset{˜}{H}}^{1/2}(\Gamma )$, the Lax–Milgram lemma provides existence and uniqueness of the solution *u*.

### 2.2 Model problem on closed boundaries

Assume that Γ=∂Ω is closed. Then, *W* is a linear and bounded operator from $H^{1/2}(\partial \Omega )$ to $H_{⋆}^{-1/2}(\partial \Omega )\;≔\{\psi \in H^{-1/2}(\partial \Omega ):{\langle \psi ,1\rangle }_{L^{2}(\partial \Omega )}=0\}$. Moreover, *W* is elliptic on the subspace $H^{1/2}(\partial \Omega )/R\equiv H_{⋆}^{1/2}(\partial \Omega )\;≔\{v\in H^{1/2}(\partial \Omega ):\int_{\partial \Omega }v\;d\Gamma =0\}$, where connectedness of ∂Ω is required. Let $f\in H_{⋆}^{-1/2}(\partial \Omega )$. The variational form of (8) now reads

(10) $${\langle \mathrm{Wu},v\rangle }_{L^{2}(\partial \Omega )}={\langle f,v\rangle }_{L^{2}(\partial \Omega )}\;\mathrm{for}\;\mathrm{all}\;v\in H_{⋆}^{1/2}(\partial \Omega ).$$

As before, the left-hand side defines a scalar product on $H_{⋆}^{1/2}(\partial \Omega )$, and the Lax–Milgram lemma thus provides existence and uniqueness of the solution *u*.

We note that, for certain right-hand sides *f* and Γ=∂Ω, (8) is an equivalent formulation of the Neumann problem

(11) $$-\Delta u=f\;\mathrm{in}\;\Omega ,\;\partial_{n}u=g\;\mathrm{on}\;\partial \Omega .$$

In this case, the solution *u* of (8) is, up to some additive constant, the trace $u{|}_{\partial \Omega }$ of the solution u of (11).

### 2.3 Galerkin boundary element discretization

Let $T_{h}$ be a partition of Γ into affine line segments. Let $S^{1}(T_{h})$ denote the space of all functions *v*_*h*_ which are continuous and $T_{h}-\mathrm{piecewise}$ affine with respect to the arclength. For Γ⫋∂Ω, $S_{0}^{1}(T_{h})\;≔S^{1}(T_{h})\cap {\overset{˜}{H}}^{1/2}(\Gamma )$ denotes the subspace of all functions $v_{h}\in S^{1}(T_{h})$ which additionally vanish at the tips of Γ. For Γ=∂Ω, $S_{0}^{1}(T_{h})\;≔S^{1}(T_{h})\cap H_{⋆}^{1/2}(\Gamma )$ denotes the subspace of all functions $v_{h}\in S^{1}(T_{h})$ which satisfy $\int_{\Gamma }v_{h}\;d\Gamma =0$. In either case, $S_{0}^{1}(T_{h})$ is a conforming subspace of ${\overset{˜}{H}}^{1/2}(\Gamma )$ resp. $H_{⋆}^{1/2}(\partial \Omega )$. In particular, the Galerkin formulation of (9) resp. (10) reads

(12) $${\langle {\mathrm{Wu}}_{h},v_{h}\rangle }_{L^{2}(\Gamma )}={\langle f,v_{h}\rangle }_{L^{2}(\Gamma )}\;\mathrm{for}\;\mathrm{all}\;v_{h}\in S_{0}^{1}(T_{h})$$

and admits a unique Galerkin solution $u_{h}\in S_{0}^{1}(T_{h})$.

### 2.4 ZZ-type error estimator

Let $h\in L^{\infty }(\Gamma )$ be the local mesh-size function defined by

(13) $$h{|}_{T}\;≔\mathrm{length}(T)\;\mathrm{for}\;T\in T_{h}$$

with the arclength length(·). With (·)′ denoting the arclength derivative, we propose the following ZZ-type error estimator

(14) $$\eta_{h}=\|h^{1/2}(1-A_{h})u_{h}^{\prime }{\|}_{L^{2}(\Gamma )},$$

where $A_{h}:L^{2}(\Gamma )\to S^{1}(T_{h})$ denotes the Clément operator defined by

(15) $$(A_{h}v)(z)\;≔\frac{1}{\mathrm{length}(\omega_{z})}\int_{\omega_{z}}v\;d\Gamma$$

for all nodes $z\in K_{h}$ of $T_{h}$ with $\omega_{z}=⋃\{T\in T_{h}:z\in T\}$ the node patch.

### 2.5 Adaptive mesh-refining algorithm

Given a right-hand side $f\in H^{-1/2}(\Gamma )$, an initial partition $T_{h}$ of Γ, and some adaptivity parameter 0<θ<1, the proposed adaptive algorithm reads as follows:

(i) Compute discrete solution $u_{h}\in S_{0}^{1}(T_{h})$.
(ii) For all $T\in T_{h}$, compute the refinement indicators
  (16) $$\eta_{h}{\left(T\right)}^{2}\;≔\mathrm{length}(T)\|(1-A_{h})u_{h}^{\prime }{\|}_{L^{2}(T)}^{2}.$$
(iii) Determine a set $M_{h}\subseteq T_{h}$ such that
  (17) $$\theta \eta_{h}^{2}\leq \underset{T\in M_{h}}{\sum }\eta_{h}{\left(T\right)}^{2}.$$
(iv) Generate a new mesh $T_{h}$ by bisection of at least all elements in $M_{h}$.
(v) Goto (i) and iterate.

For the proof of quasi-optimal convergence rates in the frame of adaptive FEM, e.g., [44,13], and adaptive BEM [28,47], the set $M_{h}$ in step (iii) is usually chosen with minimal cardinality. A greedy algorithm sorts the indicators in descending order and then iteratively splits $T_{h}$ into $M_{h}$ and $T_{h}â§¹M_{h}$ by moving the largest indicator from $T_{h}â§¹M_{h}$ to $M_{h}$ until the Dörfler criterion (17) is satisfied.

For our implementation, we use the Matlab BEM library HILBERT [1]. The local mesh-refinement in step (iv) of the algorithm is done by some bisection-based algorithm from [2] which guarantees that the local mesh-ratio

(18) $$\kappa (T_{h})\;≔\max\{\frac{\mathrm{length}(T)}{\mathrm{length}(T\prime )}:T,T\prime \in T_{h}\;\mathrm{neighbors}\}$$

stays uniformly bounded $\kappa (T_{h})\leq \gamma$ for some γ≥2 which depends only on the initial partition. We stress that such a property is required for the numerical analysis of *η*_*h*_ in Sections 5 and 6 below.

We recall from the literature [42] that the optimal rate of convergence with lowest-order BEM is $O(h^{3/2})$ if the exact solution is smooth. This corresponds to $O(N^{-3/2})$ with respect to the number *N* of elements of adaptively generated meshes.

### 2.6 Numerical experiment for slit problem

We consider the hyper-singular integral equation

(19) Wu=1onΓ=(−1,1)×{0}.

The exact solution is known and reads $u(x,0)=2\sqrt{1-x^{2}}$. Note that $u\in {\overset{˜}{H}}^{1/2}(\Gamma )\cap H^{1-ɛ}(\Gamma )$ for all ɛ>0. In particular, we expect an empirical convergence order $O(h^{1/2})$ for uniform mesh-refinement.

**Fig. 1:**
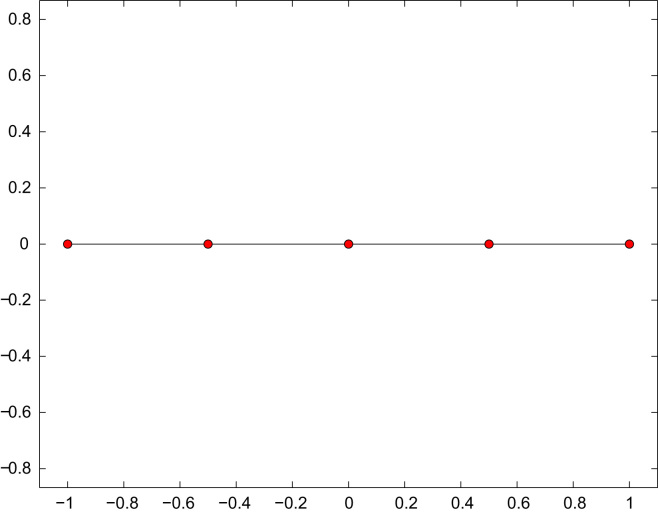
Slit Γ=(−1,1)×{0} and initial mesh $T_{h}$ with *N*=4 elements of the numerical experiment for the hyper-singular integral equation from Section 2.6 and the weakly singular integral equation from Section 3.5.

**Fig. 2:**
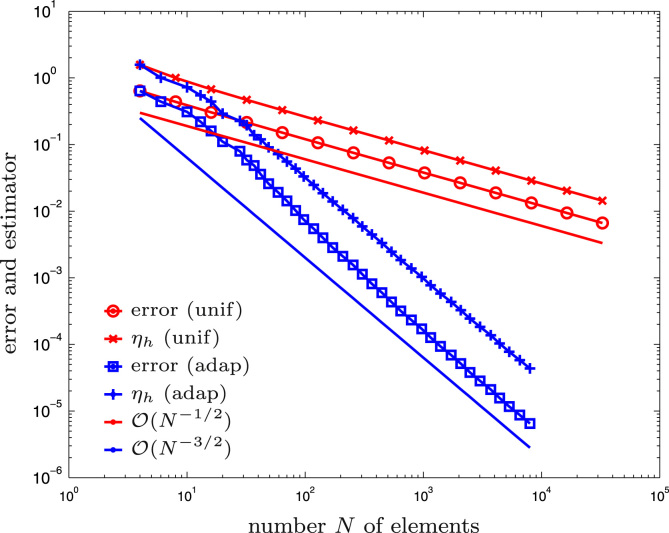
Numerical outcome of the experiment for the hyper-singular integral equation from Section 2.6 and uniform vs. adaptive mesh-refinement.

The initial mesh $T_{h}$ for the computation is shown in Fig. 1. We compare adaptive mesh-refinement with parameter θ=1/2 with uniform mesh-refinement. The corresponding convergence graphs are visualized in Fig. 2. While uniform mesh-refinement leads to the predicted suboptimal order $O(h^{1/2})=O(N^{-1/2})$, the proposed adaptive strategy regains the optimal rate $O(N^{-3/2})$.

### 2.7 Numerical experiment on closed boundary

**Fig. 3:**
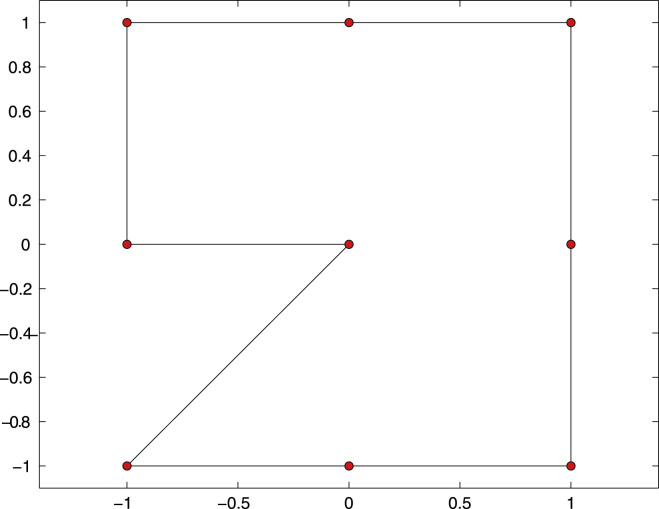
Boundary Γ=∂Ω and initial mesh $T_{h}$ with *N*=9 elements of the numerical experiment for the hyper-singular integral equation from Section 2.7.

We consider the Z-shaped domain with reentrant corner at the origin (0,0), see Fig. 3 for a sketch. The right-hand side $f=(1/2-K\prime )(\partial_{n}u)\in H^{-1/2}(\Gamma )$ with Γ=∂Ω and K′ the adjoint double layer-potential is chosen such that the hyper-singular integral equation (8) is equivalent to some Neumann problem (11) with f=0. The exact solution reads

(20) $$u(x)=r^{4/7}\;\cos(4\varphi /7)$$

in 2D polar coordinates x=r(cosφ,sinφ). The exact solution *u* of (8) is, up to some additive constant, the trace $u{|}_{\Gamma }$. Moreover, *u* admits a generic singularity at the reentrant corner. Note that $u\in H_{⋆}^{1/2}(\partial \Omega )\cap H^{4/7+1/2-ɛ}(\partial \Omega )$ for all ɛ>0. Theoretically, this predicts an expected convergence order $O(h^{4/7})$ for uniform mesh-refinement.

**Fig. 4:**
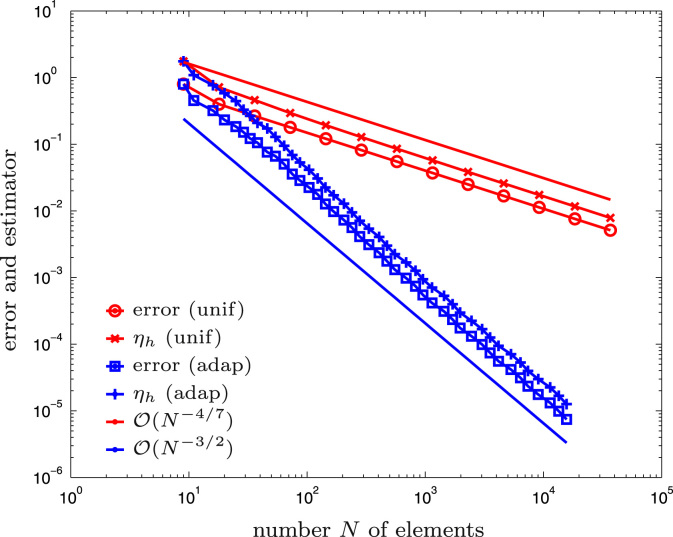
Numerical outcome of the experiment for the hyper-singular integral equation from Section 2.7 and uniform vs. adaptive mesh-refinement.

The Z-shaped domain as well as the initial mesh $T_{h}$ for the computation are shown in Fig. 3. We compare adaptive mesh-refinement with parameter θ=1/2 with uniform mesh-refinement. The corresponding convergence graphs are visualized in Fig. 4. While uniform mesh-refinement leads to the expected rate $O(h^{4/7})=O(N^{-4/7})$, the proposed adaptive strategy regains the optimal rate $O(N^{-3/2})$.

## 3 Weakly singular integral equation

In this section, we consider the simple-layer potential

(21) $$(V\phi )(x)=\int_{\Gamma }G(x-y)\phi (y)\;d\Gamma (y)\;\mathrm{for}\;x\in \Gamma ,$$

where G(·) denotes the fundamental solution of the 2D Laplacian from (6). We assume that Γ⊆∂Ω is a relatively open but possibly non-connected subset of the boundary ∂Ω and that diam(Ω)<1. For the following facts on the functional analytic setting as well as for proofs and further details, we again refer to, e.g., the monographs [33,36,42].

### 3.1 Model problem

It is well-known that *V* is a linear, bounded, and elliptic operator from ${\overset{˜}{H}}^{-1/2}(\Gamma )$ to its dual $H^{1/2}(\Gamma )$, where ellipticity follows from diam(Ω)<1. Given some $f\in H^{1/2}(\Gamma )$, we aim at the numerical solution of the weakly singular integral equation

(22) Vϕ=f.

We use the variational form

(23) $${\langle V\phi ,\psi \rangle }_{L^{2}(\Gamma )}={\langle f,\psi \rangle }_{L^{2}(\Gamma )}\;\mathrm{for}\;\mathrm{all}\;\psi \in {\overset{˜}{H}}^{-1/2}(\Gamma ).$$

The left-hand side defines an equivalent scalar product on ${\overset{˜}{H}}^{-1/2}(\Gamma )$, and the Lax–Milgram lemma thus provides existence and uniqueness of the solution $\phi \in {\overset{˜}{H}}^{-1/2}(\Gamma )$ of (23).

We stress that, for certain right-hand sides *f* and Γ=∂Ω, (22) is an equivalent formulation of the Dirichlet problem

(24) −Δu=finΩ,u=gonΓ.

In this case, it holds $\phi =\partial_{n}u$. In particular, one cannot expect that *ϕ* is locally smooth, where the outer normal vector *n* is not.

### 3.2 Galerkin boundary element discretization

Let $T_{h}$ be a partition of Γ into affine line segments. Let $P^{0}(T_{h})$ denote the space of all $T_{h}-\mathrm{piecewise}$ constant functions *ψ*_*h*_. For the Galerkin discretization, we replace $\phi ,\psi \in {\overset{˜}{H}}^{-1/2}(\Gamma )$ by discrete functions $\phi_{h},\psi_{h}\in P^{0}(T_{h})$. Then, $P^{0}(T_{h})\subset {\overset{˜}{H}}^{-1/2}(\Gamma )$ is a conforming subspace, and the Galerkin formulation

(25) $${\langle V\phi_{h},\psi_{h}\rangle }_{L^{2}(\Gamma )}={\langle f,\psi_{h}\rangle }_{L^{2}(\Gamma )}\;\mathrm{for}\;\mathrm{all}\;\psi_{h}\in P^{0}(T_{h})$$

admits a unique Galerkin solution $\phi_{h}\in P^{0}(T_{h})$.

### 3.3 ZZ-type error estimator

With $h\in L^{\infty }(\Gamma )$ the local mesh-size function from (13), we propose the following ZZ-type error estimator:

(26) $$\eta_{h}=\|h^{1/2}(1-A_{h})\phi_{h}{\|}_{L^{2}(\Gamma )}.$$

As noted before, we may expect that *ϕ* is non-smooth at points x∈Γ, where the normal mapping x↦n(x) is non-smooth. Therefore, we slightly modify the Clément operator $A_{h}:L^{2}(\Gamma )\to P^{1}(T_{h})$ from (15) as follows:

• First, if $\{z\}=T_{j}\cap T_{k}$ is the node between the elements $T_{j},T_{k}\in T_{h}$ and if the normal vector of *T*_*j*_ and *T*_*k*_ does not jump at *z*, we define
  (27) $$(A_{h}v)(z)\;≔\frac{1}{\mathrm{length}(\omega_{z})}\int_{\omega_{z}}v\;d\Gamma$$
  with $\omega_{z}=⋃\{T\in T_{h}:z\in T\}=T_{j}\cup T_{k}$ the node patch.
• Second, if the normal vectors of *T*_*j*_ and *T*_*k*_ differ at *z*, we allow $A_{\mathrm{hv}}$ to jump at *z* as well, namely
  (28) $$(A_{h}v){|}_{T_{j}}(z)=\frac{1}{\mathrm{length}(T_{j})}\int_{T_{j}}v\;d\Gamma ,(A_{h}v){|}_{T_{k}}(z)=\frac{1}{\mathrm{length}(T_{k})}\int_{T_{k}}v\;d\Gamma .$$

Note that this definition can only be meaningful if each connected component γ⊆Γ on which the normal mapping x↦n(x) is smooth consists of at least two elements. Otherwise, $\gamma =T_{j}$ would lead to $\phi_{h}{|}_{\gamma }=(A_{h}\phi_{h}){|}_{\gamma }$ so that *η*_*h*_ vanishes on *γ*, i.e., *T*_*j*_ would never be marked for refinement by an adaptive algorithm.

### 3.4 Adaptive algorithm

We consider the adaptive algorithm from Section 2.5 with the obvious modifications, i.e., we compute $\phi_{h}\in P^{0}(T_{h})$ in step (i) as well as the local contributions

(29) $$\eta_{h}{\left(T\right)}^{2}\;≔\mathrm{length}(T)\|(1-A_{h})\phi_{h}{\|}_{L^{2}(T)}^{2}$$

in step (ii). We refer to the literature, e.g., [42], that the optimal rate of lowest-order BEM is $O(h^{3/2})$ for a smooth solution *ϕ*, and the adaptive algorithm thus aims to regain a convergence order $O(N^{-3/2})$ with respect to the number of elements.

### 3.5 Numerical experiment for slit problem

We consider the weakly singular integral equation

(30) Vϕ=1onΓ=(−1,1)×{0}.

The unique exact solution of this equation is known and reads $\phi (x,0)=-2x/\sqrt{1-x^{2}}$. Note that $\phi \in {\overset{˜}{H}}^{-1/2}(\Gamma )\cap H^{-ɛ}(\Gamma )$ for all ɛ>0. In particular, we expect an empirical convergence order $O(h^{1/2})$ for uniform mesh-refinement.

**Fig. 5:**
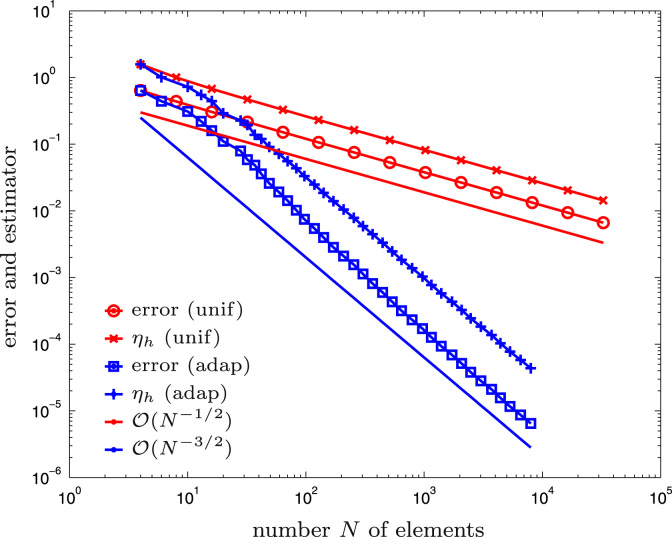
Numerical outcome of the experiment for the weakly singular integral equation from Section 3.5 and uniform vs. adaptive mesh-refinement.

The initial mesh $T_{h}$ for the computation is shown in Fig. 1. We compare adaptive mesh-refinement with parameter θ=1/2 with uniform mesh-refinement. The corresponding convergence graphs are visualized in Fig. 5. While uniform mesh-refinement leads to the expected rate $O(h^{1/2})=O(N^{-1/2})$, the adaptive algorithm regains the optimal rate $O(N^{-3/2})$.

### 3.6 Numerical experiment on closed boundary

**Fig. 6:**
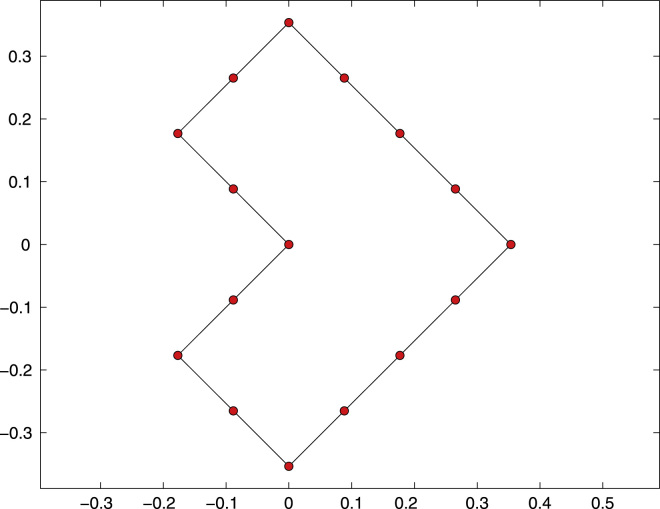
Boundary Γ=∂Ω and initial mesh $T_{h}$ with *N*=16 elements of the numerical experiment from Section 3.6.

We consider the rotated L-shaped domain Ω from Fig. 6 with reentrant corner at the origin (0,0) and boundary Γ=∂Ω. We choose the right-hand side $f=(K+1/2)(u{|}_{\Gamma })\in H^{1/2}(\Gamma )$ with *K* the double-layer potential, so that the weakly singular integral equation (22) is equivalent to some Dirichlet problem (24) with f=0. The exact solution of (24) is prescribed as

(31) $$u(x)=r^{2/3}\;\cos(2\varphi /3)$$

in 2D polar coordinates x=r(cosφ,sinφ) and admits a generic singularity at the reentrant corner. The exact solution *ϕ* of (22) is the normal derivative $\phi =\partial_{n}u$. We note that $\phi \in H^{2/3-1/2-ɛ}(\Gamma )$ for all ɛ>0, and we may hence expect convergence of order $O(h^{2/3})$ for uniform mesh-refinement.

**Fig. 7:**
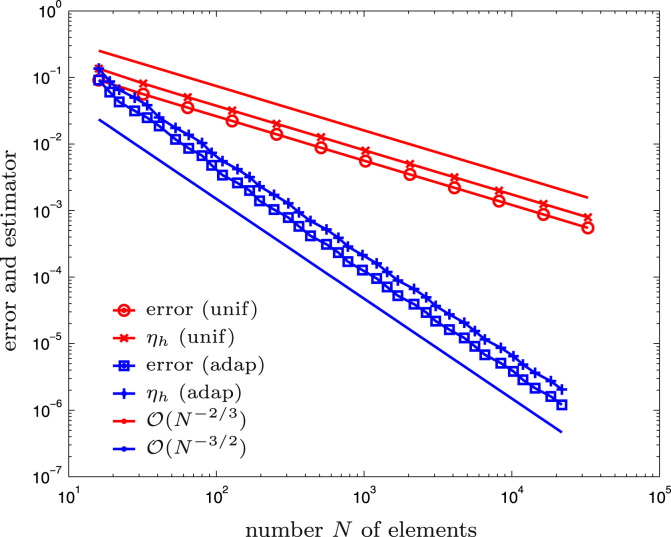
Numerical outcome of the experiment for the weakly singular integral equation from Section 3.6 and uniform vs. adaptive mesh-refinement.

The L-shaped domain as well as the initial mesh $T_{h}$ for the computation are shown in Fig. 6. We compare adaptive mesh-refinement with parameter θ=1/2 with uniform mesh-refinement. The corresponding convergence graphs are visualized in Fig. 7. The proposed adaptive algorithm recovers the optimal order of convergence.

## 4 Preliminaries

The purpose of this short section is to fix the notation of the spaces involved and to recall standard results used in the following.

### 4.1 Interpolation spaces

Let *X*_0_ and *X*_1_ be Hilbert spaces with $X_{0}⊇X_{1}$ and continuous inclusion, i.e., there exists some constant C>0 such that

(32) $$\|x{\|}_{X_{0}}\leq C\|x{\|}_{X_{1}}\;\mathrm{for}\;\mathrm{all}\;x\in X_{1}.$$

Interpolation theory, e.g., [7], provides a means to define intermediate spaces

(33) $$X_{1}\subseteq X_{s}\;≔{\left[X_{0};X_{1}\right]}_{s}\subseteq X_{0}\;\mathrm{for}\;\mathrm{all}\;0<s<1,$$

where ${\left[\cdot ;\cdot \right]}_{s}$ denotes the interpolation operator of, e.g., the *K*-method. The norm related to the intermediate interpolation space *X*_*s*_ satisfies

(34) $$\|x{\|}_{X_{s}}\leq \|x{\|}_{X_{0}}^{1-s}\|x{\|}_{X_{1}}^{s}\;\mathrm{for}\;\mathrm{all}\;x\in X_{1}.$$

The most important consequence, however, is the so-called *interpolation estimate*: Let $X_{0}⊇X_{1}$ and $Y_{0}⊇Y_{1}$ be Hilbert spaces with continuous inclusions. Let $T:X_{0}\to Y_{0}$ be a linear operator with $T(X_{1})\subseteq Y_{1}$. Assume that $T:X_{0}\to Y_{0}$ as well as $T:X_{1}\to Y_{1}$ are continuous, i.e.,

(35) $$\|\mathrm{Tx}{\|}_{Y_{0}}\leq C_{1}\|x{\|}_{X_{0}}\;\mathrm{for}\;\mathrm{all}\;x\in X_{0},\|\mathrm{Tx}{\|}_{Y_{1}}\leq C_{2}\|x{\|}_{X_{1}}\;\mathrm{for}\;\mathrm{all}\;x\in X_{1},$$

with the respective operator norms $C_{1}$, $C_{2}>0$. Let 0<s<1 and $X_{s}={\left[X_{0};X_{1}\right]}_{s}$ and $Y_{s}={\left[Y_{0};Y_{1}\right]}_{s}$. Then, $T:X_{s}\to Y_{s}$ is a well-defined linear and continuous operator with

(36) $$\|\mathrm{Tx}{\|}_{Y_{s}}\leq C_{1}^{1-s}C_{2}^{s}\|x{\|}_{X_{s}}\;\mathrm{for}\;\mathrm{all}\;x\in X_{s}.$$

Note that for other interpolation methods than the *K*-method, the previous estimates (34) and (36) hold only up to some additional generic constant which depends only on Γ, see e.g., [7].

### 4.2 Function spaces

Let $L^{2}(\Gamma )$ denote the space of square integrable functions on Γ, associated with the Hilbert norm

(37) $${\left\|v\right\|}_{L^{2}(\Gamma )}^{2}\;≔\int_{\Gamma }v^{2}\;d\Gamma .$$

Note that $\|\cdot {\|}_{L^{2}(\Gamma )}$ stems from the scalar product

(38) $${\langle v,w\rangle }_{L^{2}(\Gamma )}\;≔\int_{\Gamma }\mathrm{vw}\;d\Gamma .$$

Let $H^{1}(\Gamma )$ denote the closure of all Lipschitz continuous functions on Γ with respect to the Hilbert norm

(39) $${\left\|v\right\|}_{H^{1}(\Gamma )}^{2}\;≔{\left\|v\right\|}_{L^{2}(\Gamma )}^{2}+{\left\|v\prime \right\|}_{L^{2}(\Gamma )}^{2}.$$

Let ${\overset{˜}{H}}^{1}(\Gamma )$ denote the closure of all Lipschitz continuous functions on Γ with respect to the $H^{1}(\Gamma )-\mathrm{norm}$ which vanish at the tips of Γ. We stress that both $H^{1}(\Gamma )$ and ${\overset{˜}{H}}^{1}(\Gamma )$ are dense subspaces of $L^{2}(\Gamma )$ with respect to the $L^{2}(\Gamma )-\mathrm{norm}$. Moreover, it holds $H^{1}(\Gamma )={\overset{˜}{H}}^{1}(\Gamma )$ in case of a closed boundary Γ=∂Ω.

Sobolev spaces of fractional order 0<s<1 are defined by interpolation

(40) $$H^{s}(\Gamma )\;≔{\left[L^{2}(\Gamma );H^{1}(\Gamma )\right]}_{s},{\overset{˜}{H}}^{s}(\Gamma )\;≔{\left[L^{2}(\Gamma );{\overset{˜}{H}}^{1}(\Gamma )\right]}_{s}.$$

To abbreviate notation, we shall also write $L^{2}(\Gamma )=H^{0}(\Gamma )={\overset{˜}{H}}^{0}(\Gamma )$. It follows that all $H^{s}(\Gamma )$ and ${\overset{˜}{H}}^{s}(\Gamma )$ are dense subspaces of $L^{2}(\Gamma )$ with respect to the $L^{2}(\Gamma )-\mathrm{norm}$. Therefore, the dual spaces can be understood with respect to the extended $L^{2}(\Gamma )-\mathrm{scalar}$ product. For −1≤s<0, we define

(41) $$H^{-s}(\Gamma )\;≔{\overset{˜}{H}}^{s}{\left(\Gamma \right)}^{⁎},{\overset{˜}{H}}^{-s}(\Gamma )\;≔H^{s}{\left(\Gamma \right)}^{⁎}.$$

It follows that $L^{2}(\Gamma )$ is dense in $H^{-s}(\Gamma )$ and ${\overset{˜}{H}}^{-s}(\Gamma )$ with respect to the associated norms. For *s*=0, we let ${\overset{˜}{H}}^{0}(\Gamma )\;≔L^{2}(\Gamma )≕H^{0}(\Gamma )$.

We stress that interpolation theory also states the equalities

(42) $$H^{-s}(\Gamma )={\left[H^{-1}(\Gamma );L^{2}(\Gamma )\right]}_{s},{\overset{˜}{H}}^{-s}(\Gamma )={\left[{\overset{˜}{H}}^{-1}(\Gamma );L^{2}(\Gamma )\right]}_{s}$$

in the sense of sets and equivalent norms [36]. Moreover, interpolation reveals the continuous inclusions ${\overset{˜}{H}}^{\pm s}(\Gamma )\subseteq H^{\pm s}(\Gamma )$ as well as ${\overset{˜}{H}}^{\pm s}(\partial \Omega )=H^{\pm s}(\partial \Omega )$.

The analysis of the hyper-singular integral equation further requires

(43) $$H_{⋆}^{\pm s}(\partial \Omega )\;≔\{v\in H^{\pm s}(\partial \Omega ):{\langle v,1\rangle }_{L^{2}(\partial \Omega )}=0\}$$

for 0≤s≤1. We define $L_{⋆}^{2}(\Gamma )\;≔H_{⋆}^{0}(\Gamma )$. We again note that interpolation yields the equality

(44) $$H_{⋆}^{\pm s}(\partial \Omega )={\left[L_{⋆}^{2}(\partial \Omega );H_{⋆}^{1}(\partial \Omega )\right]}_{s}.$$

Finally, $H_{0}^{s}(\Gamma )$ denotes either ${\overset{˜}{H}}^{s}(\Gamma )$ for Γ⫋∂Ω resp. $H_{⋆}^{s}(\partial \Omega )$ for Γ=∂Ω. In either case, $H_{0}^{s}(\Gamma )$ contains no constant function different from zero provided that Γ is connected.

### 4.3 Discrete spaces

We assume that $T_{h}=\{T_{1},\ldots ,T_{N}\}$ is a partition of Γ into finitely many compact and affine line segments $T\in T_{h}$. With each element $T\in T_{h}$, we associate an affine bijection $\gamma_{T}:[0,1]\to T$.

For $q\in N_{0}$, let $P^{q}$ denote the space of polynomials of degree ≤q on R. With this, we define the space of $T_{h}-\mathrm{piecewise}$ polynomials by

(45) $$P^{q}(T_{h})\;≔\{v_{h}:\Gamma \to R:\forall T\in T\;v_{h}{}^{\circ}\gamma_{T}\in P^{q}\}.$$

Note that functions $v_{h}\in P^{q}(T_{h})$ are discontinuous in general. Special attention is paid to the piecewise constants $P^{0}(T_{h})$.

If continuity is required, we use the space

(46) $$S^{q}(T_{h})\;≔P^{q}(T_{h})\cap C(\Gamma )$$

of continuous splines of piecewise degree q≥1. Special attention is paid to the Courant space $S^{1}(T_{h})$ of lowest order.

For the treatment of the hyper-singular integral equation, we additionally define

(47) $${\overset{˜}{S}}^{q}(T_{h})\;≔S^{q}(T_{h})\cap {\overset{˜}{H}}^{1}(\Gamma ),$$

(48) $$S_{⋆}^{q}(T_{h})\;≔S^{q}(T_{h})\cap H_{⋆}^{1}(\Gamma ).$$

Finally, $S_{0}^{q}(T_{h})$ denotes either ${\overset{˜}{S}}^{q}(T_{h})$ for Γ⫋∂Ω resp. $S_{⋆}^{q}(T_{h})$ for Γ=∂Ω.

### 4.4 Projections

Let *X*_*h*_ be a finite dimensional subspace of a Hilbert space *X*. The *X*-orthogonal projection onto *X*_*h*_ is the unique linear operator $P_{h}:X\to X_{h}$ such that, for all x∈X and $x_{h}\in X_{h}$, it holds

(49) $$P_{h}x_{h}=x_{h},{\langle P_{h}x,x_{h}\rangle }_{X}={\langle x,x_{h}\rangle }_{X}.$$

This implies the Pythagoras theorem

(50) $${\left\|x\right\|}_{X}^{2}={\left\|P_{h}x\right\|}_{X}^{2}+{\left\|(1-P_{h})x\right\|}_{X}^{2}$$

and consequently reveals that $P_{h}x\in X_{h}$ is the best approximation of *x* in *X*_*h*_, i.e.,

(51) $$\|(1-P_{h})x{\|}_{X}=\underset{x_{h}\in X_{h}}{\min}\|x-x_{h}{\|}_{X}.$$

In [46], a quasi-interpolation operator $J_{h}^{\Omega }:H^{1}(\Omega )\to S^{1}(T_{h}^{\Omega })$ is introduced. Here, $\Omega \subset R^{d}$ for d≥2 is a Lipschitz domain, $T_{h}^{\Omega }$ is a conforming triangulation of Ω into simplices, and $S^{1}(T_{h}^{\Omega })$ is the lowest-order Courant finite element space. It is shown that $J_{h}^{\Omega }$ has a local first-order approximation property and is a linear and continuous projection onto $S^{1}(T_{h}^{\Omega })$. Moreover, $J_{h}^{\Omega }$ preserves discrete boundary data, since the boundary values $(J_{h}v){|}_{\Gamma }$ depend only on the trace $v{|}_{\Gamma }$ with Γ=∂Ω.

Let $T_{h}$ denote the partition of Γ induced by $T_{h}^{\Omega }$. Then, the mentioned properties of $J_{h}$ yield that the restriction $J_{h}\;≔J_{h}^{\Omega }(\cdot ){|}_{\Gamma }:H^{1/2}(\Gamma )\to S^{1}(T_{h})$ to the trace space $H^{1/2}(\Gamma )$ yields a well-defined, linear, and continuous projection onto $S^{1}(T_{h})$ with respect to the $H^{1/2}(\Gamma )-\mathrm{norm}$. For an element $T\in T_{h}$, we denote by

(52) $$\omega_{T}\;≔⋃\{T\prime \in T_{h}:T\cap T\prime \neq \emptyset \}$$

its patch, i.e., the union of *T* and its (at most two) neighbors.

The original arguments in [46] for the domain-based Sobolev space $H^{1}(\Omega )$ also transfer to the Sobolev space $H^{1}(\Gamma )$ on the boundary, see also the discussion in [3]. This way, we see that $J_{h}$ has the following properties which shall be used in the analysis below:

(i) $J_{h}v$ is well-defined for all $v\in L^{2}(\Gamma )$.
(ii) $(J_{h}v){|}_{T}$ depends only on the function values $v{|}_{\omega_{T}}$ on the patch of $T\in T_{h}$.
(iii) $J_{h}$ is locally *L*^2^-stable, for all $v\in L^{2}(\Gamma )$,
  (53) $$\|(1-J_{h})v{\|}_{L^{2}(T)}\leq C_{3}\|v{\|}_{L^{2}(\omega_{T})}.$$
(iv) $J_{h}$ is locally *H*^1^-stable, for all $v\in H^{1}(\Gamma )$,
  (54) $$\|((1-J_{h})v)\prime {\|}_{L^{2}(T)}\leq C_{3}\;\|v\prime {\|}_{L^{2}(\omega_{T})}.$$
(v) $J_{h}$ has a first-order approximation property, for all $v\in H^{1}(\Gamma )$,
  (55) $$\|(1-J_{h})v{\|}_{L^{2}(T)}\leq C_{3}\|\mathrm{hv}\prime {\|}_{L^{2}(\omega_{T})}.$$
(vi) The constant $C_{3}>0$ depends only on the local mesh-ratio $\kappa (T_{h})$.

Since $\omega_{T}$ consists of at most three elements, the ℓ_2_-sums of the estimates (53)–(55) also provide global estimates with *T* and $\omega_{T}$ replaced by Γ. From (iii), we thus see that $J_{h}\in L(L^{2}(\Gamma );L^{2}(\Gamma ))$. The combination of (iii)–(iv) yields $J_{h}\in L(H^{1}(\Gamma );H^{1}(\Gamma ))$. In particular, the interpolation estimate (36) provides $J_{h}\in L(H^{s}(\Gamma );H^{s}(\Gamma ))$, for all 0≤s≤1.

## 5 A posteriori error analysis

In this section, we show that under appropriate assumptions, the ZZ-type error estimators proposed provide an upper bound for the error (*reliability*) and, up to some higher-order terms, also a lower bound for the error (*efficiency*). Our analysis builds on equivalence of seminorms on finite dimensional spaces and scaling arguments. The elementary, but abstract result employed reads as follows: if *X* is a finite dimensional space with seminorms $|\cdot {|}_{1}$ and $|\cdot {|}_{2}$, an estimate of the type

(56) $$|x{|}_{1}\leq C|x{|}_{2}\;\mathrm{for}\;\mathrm{all}\;x\in X$$

and some independent constant C>0 is equivalent to the inclusion

(57) $$\left\{x\in X:|x{|}_{2}=0\}\subseteq \{x\in X:|x{|}_{1}=0\right\}$$

of the respective null spaces. This result is used for polynomial spaces on *element patches*. To this end, the restricted partition of the patch $\omega_{T}$ from (52) is denoted by

(58) $$T_{h}{|}_{\omega_{T}}\;≔\{T\prime \in T_{h}:T\cap T\prime \neq \emptyset \}$$

for all T∈T.

### 5.1 Hyper-singular integral equation

Recall the abbreviated notation $H_{0}^{1/2}(\Gamma )$ from Section 4.2 and note that

(59) $$|v{|}^{2}\;≔{\langle \mathrm{Wv},v\rangle }_{L^{2}(\Gamma )}$$

defines an equivalent Hilbert norm on $H_{0}^{1/2}(\Gamma )$. Because of $H^{1/2}(\partial \Omega )={\overset{˜}{H}}^{1/2}(\partial \Omega )$ even with equal norms, we can simply use the norm $\left\|\cdot {\|}_{{\overset{˜}{H}}^{1/2}(\Gamma )}≃|\cdot \right|$ throughout the section.

We start with the derivation of an upper bound. The proof relies on the assumption that $T_{h}$ is the uniform refinement of some coarser mesh $T_{2h}$ and on some saturation assumption (61). While the first assumption can easily be achieved implementationally, the latter is essentially equivalent to the assumption that the numerical scheme has reached an asymptotic regime, see [29, Section 5.2] for discussion and numerical evidence.

Theorem 1 *Let* $T_{h}$ *be the uniform refinement of some mesh* $T_{2h}$, *i.e*., *all elements* $T\in T_{2h}$ *are bisected into two sons* $T_{1},T_{2}\in T_{h}$ *of half length. Let* $u_{h}\in S_{0}^{1}(T_{h})$ *and* $u_{2h}\in S_{0}^{1}(T_{2h})$ *be the respective Galerkin solutions. Then*, *it holds*

(60) $$|u_{h}-u_{2h}|\leq C_{4}\eta_{h}$$

*with some constant* $C_{4}>0$ *which depends only on* Γ *and all possible shapes of element patches* (52). *Under the saturation assumption*

(61) $$\left|u-u_{h}|\leq C_{\mathrm{sat}}|u-u_{2h}\right|$$

*with some uniform constant* $0<C_{\mathrm{sat}}<1$, *there holds*

(62) $$C_{\mathrm{sat}}^{-1}|u-u_{h}|\leq |u-u_{2h}|\leq \frac{C_{4}}{{\left(1-C_{\mathrm{sat}}^{2}\right)}^{1/2}}\eta_{h}.$$

Proof Let $\Pi_{2h}:L^{2}(\Gamma )\to P^{0}(T_{2h})$ denote the *L*^2^-orthogonal projection onto the $T_{2h}-\mathrm{piecewise}$ constants, i.e., the piecewise integral mean operator

(63) (Π2hψ)|T^=1length(T^)∫T^ψdΓforallT^∈T2h.

According to [23], it holds that

$$|u_{h}-u_{2h}|≃\|h^{1/2}(1-\Pi_{2h})u_{h}^{\prime }{\|}_{L^{2}(\Gamma )},$$

where the hidden constants depend only on Γ and the local mesh-ratio $\kappa (T_{h})$ from (18). To prove (60), we will verify

(64) $$\|h^{1/2}(1-\Pi_{2h})u_{h}^{\prime }{\|}_{L^{2}(T)}\;≲\;\|h^{1/2}(1-A_{h})u_{h}^{\prime }{\|}_{L^{2}(\omega_{T})}$$

for all $T\in T_{h}$ in the following. Both sides of (64) define seminorms on $P^{0}(T_{h}{|}_{\omega_{T}})$, where $u_{h}^{\prime }$ is replaced by an arbitrary $\psi_{h}\in P^{0}(T_{h}{|}_{\omega_{T}})$. It thus suffices to show that $\|h^{1/2}(1-A_{h})\psi_{h}{\|}_{L^{2}(\omega_{T})}=0$ implies $\|h^{1/2}(1-\Pi_{2h})\psi_{h}{\|}_{L^{2}(T)}=0$. From $\|h^{1/2}(1-A_{h})\psi_{h}{\|}_{L^{2}(\omega_{T})}=0$ and hence $\psi_{h}=A_{h}\psi_{h}$ on $\omega_{T}$, we see that *ψ*_*h*_ is constant on $\omega_{T}$, since *ψ*_*h*_ is both $T_{h}-\mathrm{piecewise}$ constant and continuous on $\omega_{T}$. By assumption, *T* has a brother $T\prime \in T_{h}$ such that T^=T∪T′∈T2h. Moreover, the definition of the patch and T∩T′≠∅ yield T^⊆ωT. Therefore, *ψ*_*h*_ is constant on T^ so that $\psi_{h}=\Pi_{2h}\psi_{h}$ on T^. This proves $\|h^{1/2}(1-\Pi_{2h})\psi_{h}{\|}_{L^{2}(T)}=0$ and thus verifies

$$\|h^{1/2}(1-\Pi_{2h})\psi_{h}{\|}_{L^{2}(T)}\;≲\;\|h^{1/2}(1-A_{h})\psi_{h}{\|}_{L^{2}(\omega_{T})}$$

for all $T\in T_{h}$ and $\psi_{h}\in P^{0}(T_{h})$. Finally, a scaling argument proves that the hidden constant depends only on the shape of the patch $\omega_{T}$. We note that each element $T\prime \in T_{h}$ is contained in at most three patches. Taking the ℓ_2_-sum in (64) over all elements $T\in T_{h}$, we arrive at

(65) $$\|h^{1/2}(1-\Pi_{2h})\psi_{h}{\|}_{L^{2}(\Gamma )}\;≲\;\|h^{1/2}(1-A_{h})\psi_{h}{\|}_{L^{2}(\Gamma )}$$

for all $\psi_{h}\in P^{0}(T_{h})$. Plugging in $\psi_{h}=u\prime_{h}$, we conclude the proof of (60).

The proof of (62) follows from abstract principles. According to the Galerkin orthogonality

$${\langle W(u-u_{h}),v_{h}\rangle }_{L^{2}(\Gamma )}=0\;\mathrm{for}\;\mathrm{all}\;v_{h}\in S_{0}^{1}(T_{h}),$$

we obtain a Pythagoras theorem for the induced Hilbert norm

$$|u-u_{h}{|}^{2}+|u_{h}-u_{2h}{|}^{2}=|u-u_{2h}{|}^{2},$$

where we use $v_{h}=u_{h}-u_{2h}$. Together with the saturation assumption (61), this results in

$$C_{\mathrm{sat}}^{-1}|u-u_{h}|\leq |u-u_{2h}|\leq \frac{1}{{\left(1-C_{\mathrm{sat}}^{2}\right)}^{1/2}}|u_{h}-u_{2h}|,$$

and (62) follows. □

Remark 2 With the same techniques as in the proof of Theorem 1, one can prove that the ZZ-type error estimator *η*_*h*_ is an upper bound for the estimator *μ*_*h*_ from [18] which is based on averaging on large patches. The analysis then requires that $T_{h}$ is a refinement of a coarser mesh $T_{\mathrm{kh}}$ for some k≥2 which depends only on Γ. Then, the saturation assumption (61) is formally avoided. However, the parameter *k* is still unknown, although *k*=2 empirically appears to be sufficient, see e.g., the numerical experiments in [18]. Moreover, the upper bound (62) holds only up to some additional best approximation error

$$\left|u-u_{h}|\;≲\;\eta_{h}+\underset{U_{h}\in S_{0}^{2}(T_{\mathrm{kh}})}{\min}|u-U_{h}\right|$$

with higher-order elements $S_{0}^{2}(T_{\mathrm{kh}})\;≔P^{2}(T_{\mathrm{kh}})\cap {\overset{˜}{H}}^{1/2}(\Gamma )\subseteq H^{1}(\Gamma )$ which are piecewise quadratic and globally continuous. If the exact solution *u* is smooth or if the mesh is appropriately graded to the singularities of *u*, this additional term is of higher-order. The reader is referred to [17] for further discussions. □

We next prove the lower bound. Unlike the reliability estimate (62), the following efficiency estimate (66) does not rely on the saturation assumption (61), but holds only up to some further best approximation error with higher-order elements. If the exact solution *u* of (8) is smooth or if the mesh is properly adapted to the singularities of *u*, this term becomes a higher-order term.

Let $S^{2,1}(T_{h})\;≔P^{2}(T_{h})\cap C^{1}(\Gamma )$ denote the set of all $T_{h}-\mathrm{piecewise}$ quadratic polynomials *p* such that *p* as well as its derivative p′ are continuous. With $S_{0}^{2,1}(T_{h})\;≔S^{2,1}(T_{h})\cap H_{0}^{1/2}(\Gamma )$, our efficiency result then reads as follows:

Theorem 3 *It holds*

(66) $$C_{5}^{-1}\eta_{h}\leq |u-u_{h}|+\underset{U_{h}\in S_{0}^{2,1}(T_{h})}{\min}|u-U_{h}|.$$

*The constant* $C_{5}>0$ *depends only on* Γ *and all possible shapes of element patches* (52).

The proof requires the following probably well-known lemma. For the convenience of the reader, we include the proof also here.

Lemma 4 *For* 0≤s≤1, *the arc-length derivative induces linear and continuous operators* $\left(\cdot )\prime :H^{s}(\Gamma )\to H^{s-1}(\Gamma \right)$ *and* $\left(\cdot )\prime :{\overset{˜}{H}}^{s}(\Gamma )\to {\overset{˜}{H}}^{s-1}(\Gamma \right)$.

Proof For *s*=1, it holds

$$\left\|v\prime {\|}_{L^{2}(\Gamma )}\leq \|v{\|}_{H^{1}(\Gamma )}\;\mathrm{for}\;\mathrm{all}\;v\in H^{1}(\Gamma \right)$$

and, by integration by parts,

$${\langle v\prime ,w\rangle }_{L^{2}(\Gamma )}=-{\langle v,w\prime \rangle }_{L^{2}(\Gamma )}\leq \|v{\|}_{L^{2}(\Gamma )}\|w{\|}_{H^{1}(\Gamma )}$$

for all $w\in {\overset{˜}{H}}^{1}(\Gamma )$. Note that here we require either that Γ=∂Ω or that *w* (or *v*) vanishes at the tips of Γ. By definition of the duality $H^{-1}(\Gamma )={\overset{˜}{H}}^{1}{\left(\Gamma \right)}^{⁎}$, this yields

$$\|v\prime {\|}_{H^{-1}(\Gamma )}\leq \|v{\|}_{L^{2}(\Gamma )}\;\mathrm{for}\;\mathrm{all}\;v\in H^{1}(\Gamma ).$$

Since $H^{1}(\Gamma )$ is dense in $L^{2}(\Gamma )$, we obtain continuity of $\left(\cdot )\prime :L^{2}(\Gamma )\to H^{-1}(\Gamma \right)$, i.e., the last estimate holds even for all $v\in L^{2}(\Gamma )$. Finally, the interpolation estimate (36) reveals

$$\|v\prime {\|}_{H^{s-1}(\Gamma )}\leq \|v{\|}_{H^{s}(\Gamma )}\;\mathrm{for}\;\mathrm{all}\;v\in H^{s}(\Gamma ),$$

i.e., $\left(\cdot )\prime :H^{s}(\Gamma )\to H^{s-1}(\Gamma \right)$ is a linear and continuous operator, even with operator norm 1.

To prove the same statement for $\left(\cdot )\prime :{\overset{˜}{H}}^{s}(\Gamma )\to {\overset{˜}{H}}^{s-1}(\Gamma \right)$, recall the duality ${\overset{˜}{H}}^{-1}(\Gamma )=H^{1}{\left(\Gamma \right)}^{⁎}$. With $v\in {\overset{˜}{H}}^{1}(\Gamma )$ and $w\in H^{1}(\Gamma )$ all foregoing steps remain valid with nothing but the obvious modifications. □

Proof of Theorem 3 Let $J_{h}:L^{2}(\Gamma )\to S^{1}(\Gamma )$ denote the Scott–Zhang projection from Section 4.4. We first show that

(67) $$\|h^{1/2}(1-A_{h})\psi_{h}{\|}_{L^{2}(\Gamma )}≲\;\|h^{1/2}(1-J_{h})\psi_{h}{\|}_{L^{2}(\Gamma )}$$

for all $\psi_{h}\in P^{0}(T_{h})$. To that end, we use a seminorm argument on $P^{0}(T_{h}{|}_{\omega_{T}})$: from $\|h^{1/2}(1-J_{h})\psi_{h}{\|}_{L^{2}(\omega_{T})}=0$, it follows that *ψ*_*h*_ is constant on $\omega_{T}$. By definition (15) of $A_{h}$ this yields $A_{h}\psi_{h}=\psi_{h}$ on *T*. Therefore, we see $\|h^{1/2}(1-A_{h})\psi_{h}{\|}_{L^{2}(T)}=0$, and

$$\|h^{1/2}(1-A_{h})\psi_{h}{\|}_{L^{2}(T)}\;≲\;\|h^{1/2}(1-J_{h})\psi_{h}{\|}_{L^{2}(\omega_{T})}$$

follows. A scaling argument proves that the hidden constant depends only on the shape of the patch $\omega_{T}$. Taking the ℓ_2_-sum of the last estimate over all elements $T\in T_{h}$, we obtain (67).

Second, we show that

(68) $$\|h^{1/2}(1-J_{h})\psi_{h}{\|}_{L^{2}(\Gamma )}\;≲\;\|\psi_{h}-\Psi_{h}{\|}_{{\overset{˜}{H}}^{-1/2}(\Gamma )}$$

for all $\psi_{h}\in P^{0}(T_{h})$ and $\Psi_{h}\in S^{1}(T_{h})$. Since the Scott–Zhang projection is stable with respect to the $h^{1/2}-\mathrm{weighted}$ *L*^2^-norm, see Section 4.4, the projection property of *J*_*h*_ gives

$$\|h^{1/2}(1-J_{h})\psi_{h}{\|}_{L^{2}(\Gamma )}=\|h^{1/2}(1-J_{h})(\psi_{h}-\Psi_{h}){\|}_{L^{2}(\Gamma )}≲\;\|h^{1/2}(\psi_{h}-\Psi_{h}){\|}_{L^{2}(\Gamma )}.$$

The inverse estimate of [30, Theorem 3.6] then concludes the proof of (68).

Finally, let $P_{h}:H_{0}^{1/2}(\Gamma )\to S_{0}^{2,1}(T_{h})$ denote the $H_{0}^{1/2}(\Gamma )-\mathrm{orthogonal}$ projection onto $S_{0}^{2,1}(T_{h})$ with respect to the energy norm |·|. Combining norm equivalence $|\cdot |≃\|\cdot {\|}_{{\overset{˜}{H}}^{1/2}(\Gamma )}$ with the estimates (67) and (68) for $\psi_{h}=u_{h}^{\prime }$ and $\Psi_{h}=(P_{h}u_{h})\prime$, we obtain

$$\|h^{1/2}(1-A_{h})u_{h}^{\prime }{\|}_{L^{2}(\Gamma )}\;≲\;\|{\left(u_{h}-P_{h}u_{h}\right)}^{\prime }{\|}_{{\overset{˜}{H}}^{-1/2}(\Gamma )}≲\;\|(1-P_{h})u_{h}{\|}_{{\overset{˜}{H}}^{1/2}(\Gamma )}≃|(1-P_{h})u_{h}|.$$

The triangle inequality and stability of $P_{h}$ yield

$$|(1-P_{h})u_{h}|\leq |(1-P_{h})u|+|u-u_{h}|.$$

Since $P_{h}u$ is the best approximation (51) of *u* in $S_{0}^{2,1}(T_{h})$ with respect to |·|, this proves (66). □

### 5.2 Weakly singular integral equation

We stress that the same results hold as for the hyper-singular integral equation. By

(69) $$|w{|}^{2}\;≔{\langle \mathrm{Vw},w\rangle }_{L^{2}(\Gamma )},$$

we now denote the Hilbert norm which is induced by the weakly singular integral operator, and note that $|\cdot |≃\|\cdot {\|}_{{\overset{˜}{H}}^{-1/2}(\Gamma )}$ is an equivalent norm on ${\overset{˜}{H}}^{-1/2}(\Gamma )$. The reliability result reads as follows:

Theorem 5 *Let* $T_{h}$ *be the uniform refinement of some mesh* $T_{2h}$, *i.e.*, *all elements* $T\in T_{2h}$ *are bisected into two sons* $T_{1},T_{2}\in T_{h}$ *of half length. Let* $\phi_{h}\in P^{0}(T_{h})$ *and* $\phi_{2h}\in P^{0}(T_{2h})$ *be the respective Galerkin solutions. Then*, *it holds*

(70) $$|\phi_{h}-\phi_{2h}|\leq C_{6}\eta_{h}$$

*with some constant* $C_{6}>0$ *which depends only on* Γ *and all possible shapes of element patches* (52). *Under the saturation assumption*

(71) $$\left|\phi -\phi_{h}|\leq C_{\mathrm{sat}}|\phi -\phi_{2h}\right|$$

*with some uniform constant* $0<C_{\mathrm{sat}}<1$, *there holds*

(72) $$C_{\mathrm{sat}}^{-1}|\phi -\phi_{h}|\leq |\phi -\phi_{2h}|\leq \frac{C_{6}}{{\left(1-C_{\mathrm{sat}}^{2}\right)}^{1/2}}\;\eta_{h}.$$

Remark 6 We refer to [2], where the saturation assumption (71) is proved in the frame of the weakly singular integral equation for the Dirichlet problem (24) and $T_{2h}$ replaced by some coarser mesh $T_{\mathrm{kh}}$ with k≥2 depending only on Γ. □

Proof of Theorem 5 We adopt the notation from the proof of Theorem 1. According to [24], it holds that

$$|\phi_{h}-\phi_{2h}|≃\|h^{1/2}(1-\Pi_{2h})\phi_{h}{\|}_{L^{2}(\Gamma )},$$

where the hidden constants depend only on Γ and the local mesh-ratio $\kappa (T_{h})$ from (18). Recall that the operator $A_{h}$ is now slightly different to the case of the hyper-singular integral equation. However, the same arguments as in the proof of Theorem 1 show that (65) remains valid. As before the hidden constant involved depends on all possible shapes of element patches in $T_{h}$. This yields (70), and (72) follows as before. □

We next prove the lower bound. As before, the following efficiency estimate (73) does not rely on the saturation assumption (71), but holds only up to some further best approximation error with higher-order elements.

Theorem 7 *It holds*

(73) $$C_{7}^{-1}\eta_{h}\leq |\phi -\phi_{h}|+\underset{\Phi_{h}\in S^{1}(T_{h})}{\min}|\phi -\Phi_{h}|.$$

*The constant* $C_{7}>0$ *depends only on* Γ *and all possible shapes of element patches* (52).

Proof Arguing along the lines of the proof of Theorem 3, we see that

$$\|h^{1/2}(1-A_{h})\phi_{h}{\|}_{L^{2}(\Gamma )}\;≲\;\|\phi_{h}-\Psi_{h}{\|}_{{\overset{˜}{H}}^{-1/2}(\Gamma )}$$

for all $\Psi_{h}\in S^{1}(T_{h})$. Let $P_{h}:{\overset{˜}{H}}^{-1/2}(\Gamma )\to S^{1}(T_{h})$ be the orthogonal projection onto $S^{1}(T_{h})$ with respect to the energy norm |·|. With norm equivalence $|\cdot |≃\|\cdot {\|}_{{\overset{˜}{H}}^{-1/2}(\Gamma )}$ and the triangle inequality, we see for $\Psi_{h}=P_{h}\phi_{h}$

$$|\phi_{h}-\Psi_{h}|=|(1-P_{h})\phi_{h}|\leq |(1-P_{h})\phi |+|(1-P_{h})(\phi -\phi_{h})|\leq |(1-P_{h})\phi |+|\phi -\phi_{h}|.$$

Since $P_{h}\phi$ is the best approximation of *ϕ* in $S^{1}(T_{h})$ with respect to |·|, we conclude the proof. □

## 6 Adaptive mesh-refinement

In this section, we prove that the constants in the a posteriori estimates of Section 5 are uniformly bounded and that the adaptive algorithms of Sections 2.5 and 3.4 are convergent.

### 6.1 Notation

For the following analysis, we slightly change the notation for the discrete quantities. Let $T_{0}$ be the given initial partition of Γ, with which the adaptive algorithm is started. Let ℓ=0,1,2,... denote the counter for the adaptive loop, i.e., we start with ℓ=0, and ℓ↦ℓ+1 is increased in step (v) of the adaptive algorithm.

The mesh in the ℓ-th step of the adaptive loop is denoted by $T_{\ell }$. With $T_{\ell }$, we associate the local mesh-size $h_{\ell }\in L^{\infty }(\Gamma )$ defined in (13). Moreover, $u_{\ell }\in S_{0}^{1}(T_{\ell })$ resp. $\phi_{\ell }\in P^{0}(T_{\ell })$ are the corresponding discrete solutions with respective ZZ-type error estimators $\eta_{\ell }$.

Throughout, we assume that mesh-refinement is based on bisection only, i.e., refined elements are bisected into two sons of half length. In step (iv) of the adaptive algorithm, we ensure

(74) $$\kappa (T_{\ell })\leq 2\kappa (T_{0})$$

Algorithmically, this mesh-refinement is stated and analyzed in [2]. In addition to (74), the properties of the mesh-refinement necessary in current proofs of quasi-optimal convergence rates for adaptive boundary element methods [28,47] and adaptive finite element methods [13,44,45] are satisfied, i.e., the so-called *overlay estimate* and *mesh-closure estimate* are valid. Moreover, bisection and boundedness (74) of the local mesh-ratio guarantee that only a finite number of shapes of element patches (52) can occur. Therefore, the constants in the a posteriori analysis of Section 5 are uniformly bounded.

### 6.2 Hyper-singular integral equation

The proof of the following theorem follows the concept of *estimator reduction* proposed in [4] for (h−h/2)-type error estimators. We show that the ZZ-type error estimator is contractive up to some vanishing perturbation

(75) $$\eta_{\ell +1}\leq q\eta_{\ell }+\alpha_{\ell }\;\mathrm{with}\;0\leq \alpha_{\ell }\overset{\ell \to \infty }{\to }0$$

for some ℓ-independent constant 0<q<1. In the current frame, however, the proof that the perturbation $\alpha_{\ell }$ tends to zero is much more involved than in [4], since it does not only rely on the a priori convergence of Lemma 9, but also on a pointwise convergence property of the averaging operator $A_{l}$.

Theorem 8 *Let* ${\left(u_{\ell }\right)}_{\ell \in N}$ *and* ${\left(\eta_{\ell }\right)}_{\ell \in N}$ *be the sequences of discrete solutions and error estimators generated by the adaptive algorithm. Then*, *it holds estimator convergence*

(76) $$\underset{\ell \to \infty }{\lim}\eta_{\ell }=0.$$

*Provided that* $|u-u_{\ell }|\;≲\;\eta_{\ell }$, *cf*. Theorem1, *we may thus conclude* $\lim_{\ell \to \infty }u_{\ell }=u$.

The proof requires the following lemmas. The first is already found in the early work [9] and will be applied for $H=H_{0}^{1/2}(\Gamma )$ and $X_{\ell }=S_{0}^{1}(T_{\ell })$ for the hyper-singular integral equation as well as for $H={\overset{˜}{H}}^{-1/2}(\Gamma )$ and $X_{\ell }=P^{0}(T_{\ell })$ for the weakly singular integral equation.

Lemma 9 **A priori convergence of Galerkin solutions**

*Suppose that H is a Hilbert space and* ${\left(X_{\ell }\right)}_{\ell \in N}$ *is a sequence of discrete subspaces with* $X_{\ell }\subseteq X_{\ell +1}$. *For* u∈H *and* ℓ∈N, *let* $u_{\ell }\in X_{\ell }$ *be the best approximation* (51) *of u in* $X_{\ell }$. *Then*, *there exists a limit* $u_{\infty }\in H$ *such that* $\lim_{\ell \to \infty }\|u_{\infty }-u_{\ell }{\|}_{X}=0$. □

The following lemma recalls local *L*^2^-stability and first-order approximation property of the averaging operator $A_{\ell }$ used.

Lemma 10 *Let* $T\in T_{\ell }$. *Then*, *the operators* $A_{\ell }:L^{2}(\Gamma )\to L^{2}(\Gamma )$ *defined in* (15) *resp*. (28) *are locally L*^2^-*stable*

(77) $$\|A_{\ell }v{\|}_{L^{2}(T)}\leq C_{8}\|v{\|}_{L^{2}(\omega_{T})},$$

*for all* $v\in L^{2}(\Gamma )$, *are locally H*^1^-*stable*

(78) $$\|(A_{\ell }v)\prime {\|}_{L^{2}(T)}\leq C_{8}\|v{\|}_{H^{1}(\omega_{T})},$$

*for all* $v\in H^{1}(\Gamma )$, *and have a local first-order approximation property*

(79) $$\|(1-A_{\ell })v{\|}_{L^{2}(T)}\leq C_{8}\|h_{\ell }v\prime {\|}_{L^{2}(\omega_{T})},$$

*for all* $v\in H^{1}(\Gamma )$. *Here*, $\omega_{T}$ *denotes the element patch* (52) *of* $T\in T_{\ell }$, *and* $C_{8}>0$ *depends only on* Γ *and the mesh-refinement chosen*.

Proof The proof follows as for usual Clément-type operators in finite element analysis, cf. e.g., [8,46]. Scaling arguments prove that the constants involved depend only on the shape of the element patch $\omega_{T}$. The mesh-refinement chosen guarantees that only finitely many patches occur so that these constants depend, in fact, only on the boundary Γ and the mesh-refinement strategy. □

The following proposition is more general than required for the proof of Theorem 8. However, it might be of general interest and might have further applications, since it also applies to FEM and higher dimensions even with the same proof. For the proof of Theorems 8 and 12 below, we shall only use the pointwise *L*^2^-convergence (80).

Proposition 11 **A priori convergence of averaging operators**

*Given the sequence* ${\left(T_{\ell }\right)}_{\ell \in N}$ *of adaptively generated meshes*, *let* $A_{\ell }:L^{2}(\Gamma )\to L^{2}(\Gamma )$ *be a linear operator which satisfies the local L*^2^-*stability* (77) *and the local first-order approximation property* (79). *Assume that*, *for all elements* $T\in T_{\ell }$ *and all functions* $v\in L^{2}(\Gamma )$, $(A_{\ell }v){|}_{T}$ *depends only on the function values* $v{|}_{\omega_{T}}$ *on the element patch* (52). *Then*, *there exists a linear and continuous limit operator* $A_{\infty }:L^{2}(\Gamma )\to L^{2}(\Gamma )$ *which satisfies*, *for all* $v\in L^{2}(\Gamma )$,

(80) $$\underset{\ell \to \infty }{\lim}\|(A_{\infty }-A_{\ell })v{\|}_{L^{2}(\Gamma )}=0.$$

*Suppose that the image additionally satisfies* $A_{\ell }(L^{2}(\Gamma ))\subseteq H^{1}(\Gamma )$ *for all* ℓ∈N *and that* $A_{\ell }$ *is locally H*^1^-*stable* (78). *Then*, *it holds the following*:

(i) *For all* 0≤s≤1, $A_{\infty }:H^{s}(\Gamma )\to H^{s}(\Gamma )$ *is a well-defined linear and continuous operator*.
(ii) *For all* 0≤s<1, $A_{\infty }$ *is the pointwise limit of* $A_{\ell }$, *i.e.*, *for all* $v\in H^{s}(\Gamma )$ *it holds*
  (81) $$\underset{\ell \to \infty }{\lim}\|(A_{\infty }-A_{\ell })v{\|}_{H^{s}(\Gamma )}=0.$$
(iii) *For all* $v\in H^{1}(\Gamma )$, $A_{\ell }v$ *converges weakly in* $H^{1}(\Gamma )$ *towards* $A_{\infty }v$ *as* ℓ→∞.

Proof For the proof, let $\omega_{\ell }(\gamma )\;≔⋃\{T\in T_{\ell }:T\cap \overset{¯}{\gamma }\neq \emptyset \}$ denote the patch of subsets γ⊆Γ with respect to $T_{\ell }$. We follow the ideas from [38] and define the following subsets of Γ:

$$\Gamma_{\ell }^{0}\;≔⋃\{T\in T_{\ell }:\omega_{\ell }(T)\subseteq ⋃(\overset{\infty }{\underset{j=\ell }{⋂}}T_{j})\},$$

$$\Gamma_{\ell }\;≔⋃\{T\in T_{\ell }:\mathrm{Exists}\;k\geq 0\;s.t.\;\omega_{T}\;\mathrm{is}\;\mathrm{at}\;\mathrm{least}\;\mathrm{uniformly}\;\mathrm{refined}\;\mathrm{in}\;T_{\ell +k}\},$$

$$\Gamma_{\ell }^{⁎}\;≔\Gamma â§¹(\Gamma_{\ell }\cup \Gamma_{\ell }^{0}).$$

According to [38, Corollary 4.1], it holds that

(82) $$\|h_{\ell }{\|}_{L^{\infty }(\omega_{\ell }(\Gamma_{\ell }))}≃\|h_{\ell }{\|}_{L^{\infty }(\Gamma_{\ell })}\overset{\ell \to \infty }{\to }0.$$

Let $v\in L^{2}(\Gamma )$ and ɛ>0 be arbitrary. Since $H^{1}(\Omega )$ is dense in $L^{2}(\Gamma )$, we find $v_{ɛ}\in H^{1}(\Gamma )$ such that $\|v-v_{ɛ}{\|}_{L^{2}(\Gamma )}\leq ɛ$. Due to the local *L*^2^-stability (77) and the approximation property (79) of $A_{\ell }$, we obtain

$$\|(1-A_{\ell })v{\|}_{L^{2}(\Gamma_{\ell })}\;≲\;\|(1-A_{\ell })v_{ɛ}{\|}_{L^{2}(\Gamma_{\ell })}+ɛ≲\;\|h_{\ell }\nabla v_{ɛ}{\|}_{L^{2}(\omega_{\ell }(\Gamma_{\ell }))}+ɛ.$$

According to (82), we find $\ell_{0}\in N$ such that

$$\|h_{\ell }\nabla v_{ɛ}{\|}_{L^{2}(\omega_{\ell }(\Gamma_{\ell }))}\leq \|h_{\ell }{\|}_{L^{\infty }(\omega_{\ell }(\Gamma_{\ell }))}\|\nabla v_{ɛ}{\|}_{L^{2}(\Gamma )}\leq ɛ$$

for all $\ell \geq \ell_{0}$. This proves

(83) $$\|(1-A_{\ell })v{\|}_{L^{2}(\Gamma_{\ell })}\;≲\;ɛ\;\mathrm{for}\;\ell \geq \ell_{0}.$$

Morin et al. [38, Proposition 4.2] state $|\Gamma_{\ell }^{⁎}|\to 0$ as ℓ→∞. Due to the non-concentration of Lebesgue functions, this yields

(84) $$\|v{\|}_{L^{2}(\omega_{\ell }(\Gamma_{\ell }^{⋆}))}\leq ɛ\;\mathrm{for}\;\mathrm{some}\;\ell_{1}\in N\;\mathrm{and}\;\mathrm{all}\;\ell \geq \ell_{1}.$$

Let $\ell \geq \max\{\ell_{0},\ell_{1}\}$ and k≥0. For $T\in T_{\ell }$, the definition of $(A_{\ell }v){|}_{T}$ depends only on $v{|}_{\omega_{\ell }(T)}$. By definition of $\Gamma_{\ell }^{0}$, we obtain

$$\|(A_{\ell }-A_{\ell +k})v{\|}_{L^{2}(\Gamma_{\ell }^{0})}=0.$$

With local *L*^2^-stability (77) and (84), we see

$$\|(A_{\ell }-A_{\ell +k})v{\|}_{L^{2}(\Gamma_{\ell }^{⁎})}\;≲\;\|v{\|}_{L^{2}(\omega_{\ell }(\Gamma_{\ell }^{⁎}))}+\|v{\|}_{L^{2}(\omega_{\ell +k}(\Gamma_{\ell }^{⁎}))}\leq 2\|v{\|}_{L^{2}(\omega_{\ell }(\Gamma_{\ell }^{⁎}))}\;≲\;ɛ.$$

Moreover, (83) and a triangle inequality prove

$$\|(A_{\ell }-A_{\ell +k})v{\|}_{L^{2}(\Gamma_{\ell })}\;≲\;ɛ.$$

The combination of the last three estimates yields

$$\|(A_{\ell }-A_{\ell +k})v{\|}_{L^{2}(\Gamma )}\;≲\;ɛ.$$

Altogether, ${\left(A_{\ell }v\right)}_{\ell }$ is thus a Cauchy sequence in $L^{2}(\Gamma )$ and hence convergent to some limit $A_{\infty }v\;≔\lim_{\ell }A_{\ell }v\in L^{2}(\Gamma )$. Elementary calculus predicts that this provides a well-defined linear operator $A_{\infty }:L^{2}(\Gamma )\to L^{2}(\Gamma )$, and the Banach–Steinhaus theorem even predicts continuity $A_{\infty }\in L(L^{2}(\Gamma );L^{2}(\Gamma ))$. This concludes the proof of (80).

Second, we suppose additionally that $A_{\ell }(L^{2}(\Gamma ))\subseteq H^{1}(\Gamma )$. Then, the *H*^1^-stability (78) yields that $A_{\ell }\in L^{2}(H^{1}(\Gamma );H^{1}(\Gamma ))$ are uniformly continuous operators. For $v\in H^{1}(\Gamma )$, the sequence ${\left(A_{\ell }v\right)}_{\ell }$ is hence bounded in $H^{1}(\Gamma )$ and thus admits a weakly convergent subsequence $A_{\ell_{k}}v\to w$ weakly in $H^{1}(\Gamma )$ as k→∞. The Rellich compactness theorem yields $A_{\ell_{k}}v\to w$ strongly in $L^{2}(\Omega )$. Uniqueness of limits therefore reveals $A_{\infty }v=w\in H^{1}(\Gamma )$. Iterating this argument, we see that each subsequence of $A_{\ell }v$ admits a further subsequence such that $A_{\ell_{k_{j}}}v$ converges to $A_{\infty }v\in H^{1}(\Gamma )$ weakly in $H^{1}(\Gamma )$. By elementary calculus, this implies weak convergence $A_{\ell }v\to A_{\infty }v$ in $H^{1}(\Gamma )$ of the entire sequence. Again, the Banach–Steinhaus theorem applies and proves that $A_{\infty }\in L(H^{1}(\Gamma );H^{1}(\Gamma ))$.

Third, the remaining claims follow from interpolation. The interpolation estimate (36) implies that the operator $A_{\infty }\in L(H^{s}(\Gamma );H^{s}(\Gamma ))$ is well-defined, linear, and continuous. Moreover, the estimate (34) of the interpolation norm and boundedness of weakly convergent sequences yield

$$\|(A_{\infty }-A_{\ell })v{\|}_{H^{s}(\Gamma )}\leq \|(A_{\infty }-A_{\ell })v{\|}_{L^{2}(\Gamma )}^{1-s}\|(A_{\infty }-A_{\ell })v{\|}_{H^{1}(\Gamma )}^{s}\overset{\ell \to 0}{\to }0$$

for all 0<s<1 and $v\in H^{1}(\Gamma )$. By density of $H^{1}(\Gamma )$ in $H^{s}(\Gamma )$ and stability of $A_{\ell }$, this results in pointwise convergence $\|(A_{\infty }-A_{\ell })v{\|}_{H^{s}(\Gamma )}\to 0$ for all $v\in H^{s}(\Gamma )$. □

Proof of Theorem 8 The triangle inequality shows

(85) $$\eta_{\ell +1}\leq \|h_{\ell +1}^{1/2}(1-A_{\ell })u_{\ell }^{\prime }{\|}_{L^{2}(\Gamma )}+\|h_{\ell +1}^{1/2}(1-A_{\ell +1})(u_{\ell +1}-u_{\ell })\prime {\|}_{L^{2}(\Gamma )}+\|h_{\ell +1}^{1/2}(A_{\ell +1}-A_{\ell })u_{\ell }^{\prime }{\|}_{L^{2}(\Gamma )}.$$

For the first term, we argue analogously to [4]: according to bisection, we have $h_{\ell +1}{|}_{T}=\frac{1}{2}\;h_{\ell }{|}_{T}$ for refined elements $T\in T_{\ell }â§¹T_{\ell +1}$. This gives

$$\|h_{\ell +1}^{1/2}(1-A_{\ell })u_{\ell }^{\prime }{\|}_{L^{2}(\Gamma )}^{2}\;\leq \underset{T\in T_{\ell }\cap T_{\ell +1}}{\sum }\eta_{\ell }{\left(T\right)}^{2}+\frac{1}{2}\underset{T\in T_{\ell }â§¹T_{\ell +1}}{\sum }\eta_{\ell }{\left(T\right)}^{2}\;=\eta_{\ell }^{2}-\frac{1}{2}\underset{T\in T_{\ell }â§¹T_{\ell +1}}{\sum }\eta_{\ell }{\left(T\right)}^{2}.$$

Since at least all marked elements are refined, the Dörfler marking strategy (17) in step (iii) of the adaptive algorithm yields

$$\underset{T\in T_{\ell }â§¹T_{\ell +1}}{\sum }\eta_{\ell }{\left(T\right)}^{2}\geq \underset{T\in M_{\ell }}{\sum }\eta_{\ell }{\left(T\right)}^{2}\geq \theta \eta_{\ell }^{2}.$$

Combining the last two estimates, we see

(86) $$\|h_{\ell +1}^{1/2}(1-A_{\ell })u_{\ell }^{\prime }{\|}_{L^{2}(\Gamma )}\leq {\left(1-\theta /2\right)}^{1/2}\eta_{\ell }.$$

Next, we consider the second term in (85). The local *L*^2^-stability (77) yields

$$\|h_{\ell +1}^{1/2}(1-A_{\ell +1}){\left(u_{\ell +1}-u_{\ell }\right)}^{\prime }{\|}_{L^{2}(\Gamma )}≲\|h_{\ell +1}^{1/2}{\left(u_{\ell +1}-u_{\ell }\right)}^{\prime }{\|}_{L^{2}(\Gamma )}.$$

The inverse estimate of [30, Theorem 3.6] and Lemma 4 give

$$\|h_{\ell +1}^{1/2}{\left(u_{\ell +1}-u_{\ell }\right)}^{\prime }{\|}_{L^{2}(\Gamma )}\;≲\;\|{\left(u_{\ell +1}-u_{\ell }\right)}^{\prime }{\|}_{{\overset{˜}{H}}^{-1/2}(\Gamma )}≲\;\|u_{\ell +1}-u_{\ell }{\|}_{{\overset{˜}{H}}^{1/2}(\Gamma )}≃|u_{\ell +1}-u_{\ell }|.$$

Together with the a priori convergence of Lemma 9, we thus see

(87) $$\|h_{\ell +1}^{1/2}(1-A_{\ell +1}){\left(u_{\ell +1}-u_{\ell }\right)}^{\prime }{\|}_{L^{2}(\Gamma )}\overset{\ell \to \infty }{\to }0.$$

Third, we consider the last term in (85): let ɛ>0. According to the a priori convergence of Lemma 9, there exists an index $k_{0}\in N$ such that

$$\|u_{\ell }-u_{k}{\|}_{{\overset{˜}{H}}^{1/2}(\Gamma )}≃|u_{\ell }-u_{k}|\leq ɛ\;\mathrm{for}\;\mathrm{all}\;k,\ell \geq k_{0}.$$

According to the *L*^2^-pointwise a priori convergence (80) of $A_{\ell }$ from Proposition 11, there exists an index $\ell_{0}\in N$ such that

$$\|(A_{\ell +1}-A_{\ell })u_{k_{0}}^{\prime }{\|}_{L^{2}(\Gamma )}\leq ɛ\;\mathrm{for}\;\mathrm{all}\;\ell \geq \ell_{0}.$$

Moreover, the local *L*^2^-stability (77) of the operators yields

$$\|h_{\ell +1}^{1/2}(A_{\ell +1}-A_{\ell })\psi {\|}_{L^{2}(\Gamma )}\;≲\;\|h_{\ell +1}^{1/2}\psi {\|}_{L^{2}(\Gamma )}.$$

Plugging in $\psi ={\left(u_{\ell }-u_{k_{0}}\right)}^{\prime }$, the inverse estimate from [30, Theorem 3.6] and Lemma 4 show

$$\|h_{\ell +1}^{1/2}(A_{\ell +1}-A_{\ell }){\left(u_{\ell }-u_{k_{0}}\right)}^{\prime }{\|}_{L^{2}(\Gamma )}\;≲\;\|h_{\ell +1}^{1/2}{\left(u_{\ell }-u_{k_{0}}\right)}^{\prime }{\|}_{L^{2}(\Gamma )}\;≲\;\|{\left(u_{\ell }-u_{k_{0}}\right)}^{\prime }{\|}_{{\overset{˜}{H}}^{-1/2}(\Gamma )}\;≲\;\|u_{\ell }-u_{k_{0}}{\|}_{{\overset{˜}{H}}^{1/2}(\Gamma )},$$

where the hidden constants depend only on Γ and uniform boundedness of the local mesh-ratio $\kappa (T_{\ell })$. For $\ell \geq \max\{k_{0},\ell_{0}\}$, we thus obtain

$$\|h_{\ell +1}^{1/2}(A_{\ell +1}-A_{\ell })u_{\ell }^{\prime }{\|}_{L^{2}(\Gamma )}\;≲\|\;(A_{\ell +1}-A_{\ell })u_{k_{0}}^{\prime }{\|}_{L^{2}(\Gamma )}+\|u_{\ell }-u_{k_{0}}{\|}_{{\overset{˜}{H}}^{1/2}(\Gamma )}\;≲\;2ɛ.$$

This proves

(88) $$\|h_{\ell +1}^{1/2}(A_{\ell +1}-A_{\ell })u_{\ell }^{\prime }{\|}_{L^{2}(\Gamma )}\overset{\ell \to \infty }{\to }0.$$

Altogether, (86)–(88) prove

$$\eta_{\ell +1}\leq {\left(1-\theta /2\right)}^{1/2}\eta_{\ell }+\alpha_{\ell }\;\mathrm{with}\;0\leq \alpha_{\ell }\overset{\ell \to \infty }{\to }0.$$

Since 0<θ≤1, the error estimator is thus contractive up to a zero sequence. Therefore, elementary calculus concludes (76). □

### 6.3 Weakly singular integral equation

As for the hyper-singular integral equation, we have the following convergence result for the adaptive algorithm of Section 3.4.

Theorem 12 *Let* ${\left(\phi_{\ell }\right)}_{\ell \in N}$ *and* ${\left(\eta_{\ell }\right)}_{\ell \in N}$ *be the sequences of discrete solutions and error estimators generated by the adaptive algorithm. Then*, *it holds*

(89) $$\underset{\ell \to \infty }{\lim}\eta_{\ell }=0.$$

*Provided that* $|\phi -\phi_{\ell }|\;≲\;\eta_{\ell }$, *cf.* Theorem5, *we may thus conclude* $\lim_{\ell \to \infty }\phi_{\ell }=\phi$.

Proof Since the proof follows analogously to that of Theorem 8, we only sketch the main steps: The triangle inequality proves

(90) $$\eta_{\ell +1}\leq \|h_{\ell +1}^{1/2}(1-A_{\ell })\phi_{\ell }{\|}_{L^{2}(\Gamma )}+\|h_{\ell +1}^{1/2}(1-A_{\ell +1})(\phi_{\ell +1}-\phi_{\ell }){\|}_{L^{2}(\Gamma )}+\|h_{\ell +1}^{1/2}(A_{\ell +1}-A_{\ell })\phi_{\ell }{\|}_{L^{2}(\Gamma )}.$$

By use of the Dörfler marking strategy (17) and the fact that all marked elements are bisected, we get

(91) $$\|h_{\ell +1}^{1/2}(1-A_{\ell })\phi_{\ell }{\|}_{L^{2}(\Gamma )}\leq {\left(1-\theta /2\right)}^{1/2}\eta_{\ell }.$$

The local *L*^2^-stability (77) and the inverse estimate of [30, Theorem 3.6] for $\left\|\cdot {\|}_{{\overset{˜}{H}}^{-1/2}(\Gamma )}≃|\cdot \right|$ yield

$$\|h_{\ell +1}^{1/2}(1-A_{\ell +1})(\phi_{\ell +1}-\phi_{\ell }){\|}_{L^{2}(\Gamma )}\;≲\;\|h_{\ell +1}^{1/2}(\phi_{\ell +1}-\phi_{\ell }){\|}_{L^{2}(\Gamma )}.≃|\phi_{\ell +1}-\phi_{\ell }|.$$

The a priori convergence of Lemma 9 thus gives

(92) $$\|h_{\ell +1}^{1/2}(1-A_{\ell +1})(\phi_{\ell +1}-\phi_{\ell }){\|}_{L^{2}(\Gamma )}\overset{\ell \to \infty }{\to }0.$$

The local *L*^2^-stability (77) of the operators $A_{\ell }$ and the inverse estimate from [30, Theorem 3.6] yield

$$\|h_{\ell +1}^{1/2}(A_{\ell +1}-A_{\ell })\phi_{\ell }{\|}_{L^{2}(\Gamma )}\;≲\;\|h_{\ell +1}^{1/2}(A_{\ell +1}-A_{\ell })\phi_{k_{0}}{\|}_{L^{2}(\Gamma )}\;+\|h_{\ell +1}^{1/2}(A_{\ell +1}-A_{\ell })(\phi_{\ell }-\phi_{k_{0}}){\|}_{L^{2}(\Gamma )}\;≲\;\|(A_{\ell +1}-A_{\ell })\phi_{k_{0}}{\|}_{L^{2}(\Gamma )}+|\phi_{\ell }-\phi_{k_{0}}|,$$

where the hidden constants depend only on Γ and uniform boundedness of the local mesh-ratio $\kappa (T_{\ell })$. Using the *L*^2^-pointwise a priori convergence (80) of $A_{\ell }$ from Proposition 11 for the first term and the a priori convergence of Lemma 9 for the second term, we see that

(93) $$\|h_{\ell +1}^{1/2}(A_{\ell +1}-A_{\ell })\phi_{\ell }{\|}_{L^{2}(\Gamma )}\overset{\ell \to \infty }{\to }0.$$

Altogether, (91)–(93) prove

$$\eta_{\ell +1}\leq {\left(1-\theta /2\right)}^{1/2}\eta_{\ell }+\alpha_{\ell }\;\mathrm{with}\;0\leq \alpha_{\ell }\overset{\ell \to \infty }{\to }0.$$

Therefore, the error estimator is contractive up to a zero sequence, and elementary calculus concludes (89). □
